# Endoscopic ultrasound-guided liver biopsy using a 22G fine-needle biopsy needle and slow pull technique: A tertiary center experience

**DOI:** 10.1371/journal.pone.0356373

**Published:** 2026-08-17

**Authors:** Petko Ivanov Karagyozov, Inna Krasimirova Dobreva, Ivelina Bozhanova Zhecheva

**Affiliations:** 1 Clinic of Gastroenterology, Acibadem City Clinic University Hospital Tokuda, Sofia, Bulgaria; 2 Medical University–Pleven, Pleven, Bulgaria; Al-Azhar University, EGYPT

## Abstract

**Background:**

Endoscopic ultrasound-guided fine needle biopsy of the liver (EUS-LB) has emerged as a safe technique to obtain liver tissue for diagnosis of diffuse parenchymal and focal liver diseases. This method is widely accepted as it can overcome numerous shortcomings of percutaneous and transjugular liver biopsy (LB), but the technique is not well studied yet. There is a paucity of literature demonstrating that EUS-LB provides an adequate tissue sample for histologic analysis. Data on the use of 22G fine-needle biopsy (FNB) needle for liver biopsy is scarce.

**Objective:**

The aim of this study was to review the experience of a large tertiary referral center with EUS-LB and to evaluate the efficacy and safety of the method using a 22G FNB needle and slow pull technique for diffuse parenchymal diseases and focal liver lesions.

**Methods:**

We conducted a retrospective observational study over a three-year period and included all consecutive patients who underwent EUS-LB for diffuse parenchymal disease or focal liver lesions using a 22G Franseen-tip needle and slow pull technique.

**Results:**

A total of 72 patients were included in the study, of which 32 were males and 40 were females. The median age was 56.9 years. Adequate biopsy rates, as assessed by pathologists, were achieved in all 72 patients. Malignant disease was found in 55 (76.4%) patients and benign disease in 17 (23.6%). No adverse events were observed during the first 48 hours or within a 30-day follow-up.

**Conclusions:**

Our study illustrates that EUS-guided liver biopsy using a 22G FNB needle provides an adequate specimen for histologic analysis, shorter postprocedural recovery time, and is a safe and effective alternative to conventional methods of liver biopsy. The use of real-time imaging guidance with Doppler also helps to reduce adverse events.

## Introduction

Liver biopsy (LB) is an essential diagnostic procedure for assessing hepatic pathologies, providing valuable insights into the etiology, severity, and prognosis of liver diseases. There are two main conventional methods of liver biopsy: percutaneous, under imaging guidance using ultrasound or computed tomography, and transjugular, which is less commonly used. In recent years, endoscopic ultrasound-guided liver biopsy (EUS-LB) has emerged as a minimally invasive alternative to traditional liver biopsy techniques for the diagnosis of diffuse parenchymal liver diseases and focal liver lesions.

Multiple studies have compared EUS-LB with conventional liver biopsy methods and reported comparable diagnostic yield, increased acquisition of complete portal tracts, and longer specimen length [1,2]. EUS-LB is associated with less postprocedural pain and shorter recovery time, while providing a lower risk of complications [3,4]. Innovations in needle types, needle sizes, and suction techniques have aimed at further optimizing the EUS-LB procedure.

The 19G core FNB needle has established itself as the needle type used in most centers to obtain histology from the liver; because of its larger lumen, material fragmentation has been reported to be uncommon [2,5].

The 22G fine-needle biopsy (FNB) needle is distinguishable by its cutting edges that provide histologically adequate and most often intact tissue core fragments, allowing proper immunohistochemical analysis, molecular profiling, and subsequent targeted therapies. Using a 22G needle and the slow pull technique contributes to less blood contamination and comparable diagnostic yield, but data on the use of a 22G FNB needle for liver biopsy remain scarce.

In this study, we retrospectively assessed the diagnostic yield and effectiveness of a 22G FNB needle in evaluating diffuse parenchymal and focal liver diseases.

## Methods

### Study design and patient population

This is a retrospective observational study conducted over a period of 3 years from January 2021 to September 2024. The objective was to evaluate the diagnostic utility and safety of a 22G FNB needle using the slow pull technique for EUS-LB of diffuse parenchymal processes and focal liver lesions. As this was a retrospective observational study in which all patient data were analyzed anonymously, the requirement for individual informed consent was waived by the Ethics Committee for Scientific Research of Acibadem City Clinic Tokuda University Hospital EAD (approval number 77/11.02.2026, dated 11 February 2026). Access to non-anonymized data was granted exclusively to the principal investigator (PIK), in strict adherence to the principles of confidentiality and data security, in accordance with the ethics approval. Data were accessed for research purposes from 01/03/2026 to 31/03/2026. One of the authors (PIK) is a standing member of this Ethics Committee; his role as an investigator on this study was disclosed to the committee, and the retrospective, fully anonymized nature of the study involved no direct patient contact or discretionary clinical decision-making. The committee comprises four members in total, three of whom are independent of this study.

The inclusion criteria were: adults aged 18 years or older requiring a liver biopsy for identifying the etiology of complex liver disease, staging of liver disease (metabolic-associated fatty liver disease or cirrhosis), and tissue acquisition of focal hepatic lesions. The exclusion criteria were: thrombocytopenia (platelet count < 50,000/µL), coagulopathy (International Normalized Ratio [INR] > 2.0), use of antiplatelet agents within 5 days prior to the procedure, inability to give consent for the clinical procedure, moderate-to-gross ascites, and decompensated liver disease (Child C cirrhosis).

### Procedure

**Fig 1:**
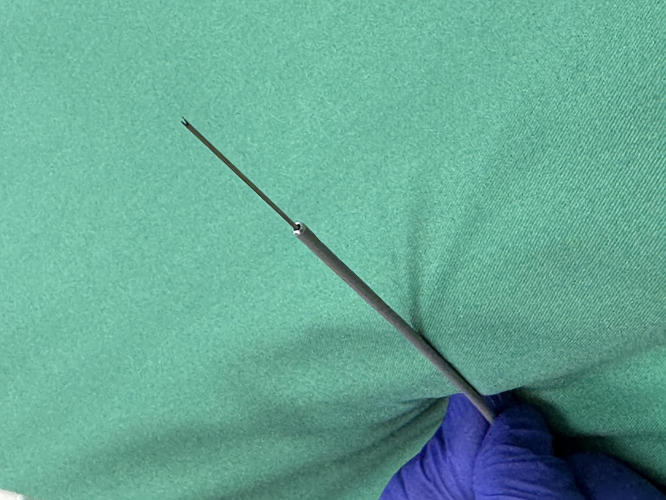
Close-up view of the 22G Franseen-tip fine-needle biopsy needle showing the crown-tip cutting edges.

**Fig 2:**
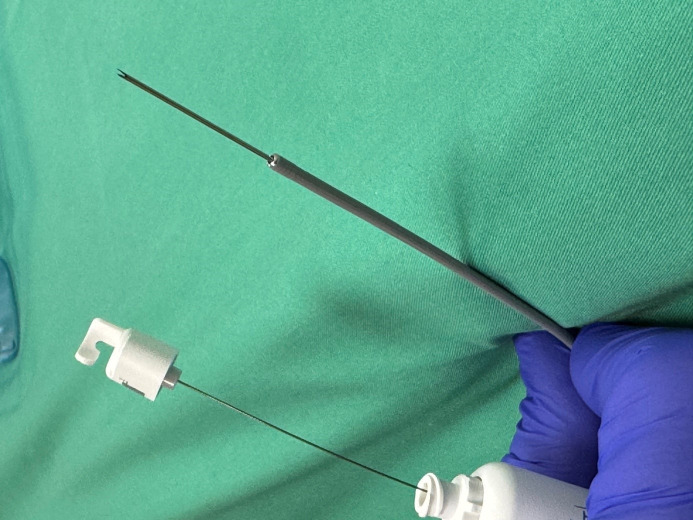
Full assembly of the 22G fine-needle biopsy system.

Endoscopic ultrasound (EUS) was performed using a linear scope (Fujifilm EG-580UT). All procedures were performed by a single endosonographer (PIK) with 6 years of dedicated EUS-FNB experience at the time of study completion. Patients were placed in the left lateral decubitus position and received procedural deep sedation with propofol administered by an anesthesiologist; no patient required general anesthesia. EUS-FNBs were obtained using a 22G tip needle (Fig 1, Fig 2). A 22G Trident™ FNB needle (Micro-Tech Endoscopy) was used in 37 patients, a 22G Acquire FNB needle (Boston Scientific Corp.) in 26 patients, and a 22G SonoTip TopGain FNB needle (MediGlobe) in 9 patients. The median number of needle passes was 3 (range 2–5).

The left lobe of the liver was accessed by a transgastric route, and the right lobe was accessed by a transduodenal route. Color Doppler imaging was used to identify vascular structures or bile ducts in the expected trajectory of the needle.

The slow pull technique was used to obtain tissue for histological analysis. The stylet was inserted into the target lesion guided by real-time EUS imaging and then was slowly withdrawn from the needle while 10–20 to-and-fro movements within the target lesion were performed. No heparinized saline flush or suction was applied, consistent with the dry slow-pull technique used throughout this study. All collected material was placed in 10% formalin and sent to the histopathology department. After completion of the procedure, the patient was observed in the recovery unit for 2 hours and returned to the clinic for ongoing care.

### Outcome measures

The primary outcome was technical success, defined as successful tissue acquisition. Secondary outcomes included specimen adequacy (total specimen length as assessed by the pathologist), diagnostic yield, and adverse event rate. As this was a retrospective, single-technique study in which only one biopsy approach was used throughout, pathologists were not blinded to the biopsy technique. Adequacy was defined as per pathologist assessment. Patients were typically discharged the following day, consistent with standard practice within the Bulgarian healthcare system. Adverse events were assessed via passive surveillance: patients were instructed to contact the clinic if they experienced any post-procedural symptoms, and adverse events were captured through chart review of any subsequent presentations during the first 48 hours and within 30 days of the procedure.

### Statistical analysis

Descriptive statistics were used to summarize patient and procedural characteristics. Continuous variables are presented as mean or median with range where appropriate. Categorical variables are presented as frequencies and percentages, with exact (Clopper-Pearson) 95% confidence intervals calculated for key proportions. All analyses were performed using standard statistical software.

## Results

**Fig 3:**
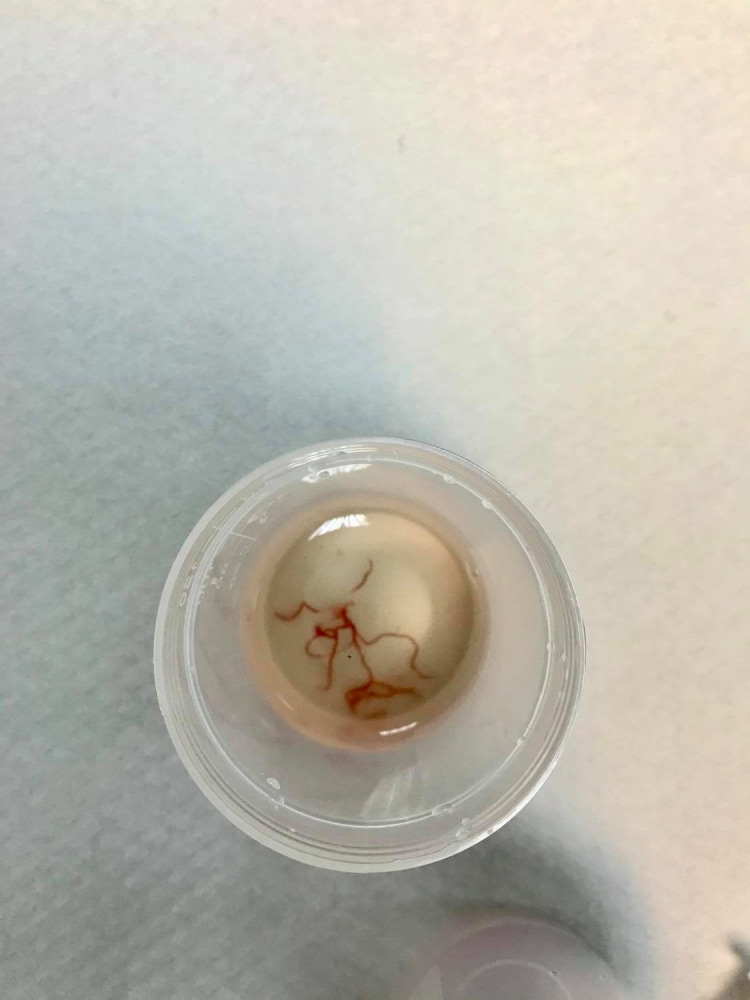
Adequate liver biopsy core specimen (~3 cm) obtained by EUS-guided FNB using the 22G Franseen-tip needle and slow pull technique.

The most common indication for biopsy was focal liver lesion or abnormal liver enzymes of unknown etiology with negative serological and imaging evaluation. A small number of patients required histological evaluation to assess the degree of fibrosis. The specimen was considered adequate in all 72 patients. The mean total length of tissue for histopathological evaluation was approximately 3 cm (Fig 3). Technical success was achieved in 100% of procedures.

We classified the EUS-FNB results into those performed for diffuse parenchymal disease and those for focal liver lesions:

EUS-FNB of diffuse parenchymal liver disease was performed in 14 (19.4%) patients: cirrhosis was found in 2 (2.7%), metabolic dysfunction-associated fatty liver disease in 6 (8.3%), and cholestasis in 6 (8.3%).

**Fig 4:**
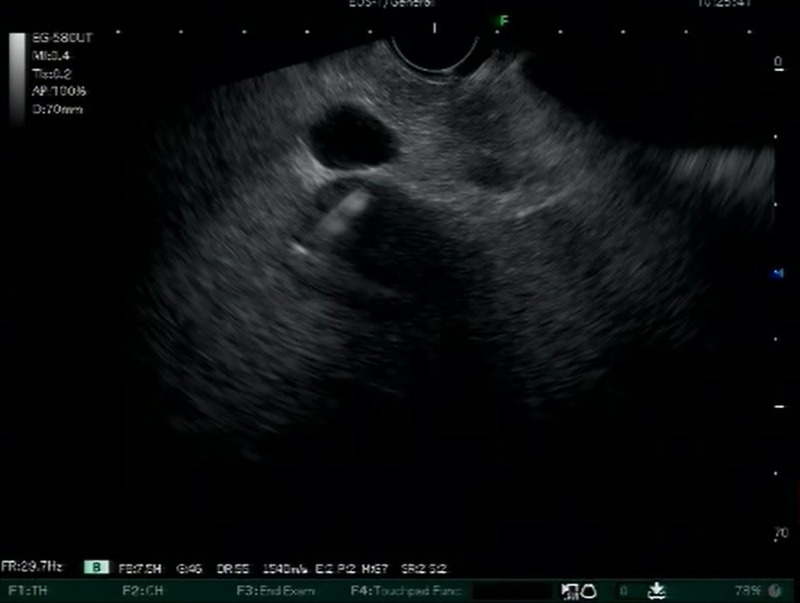
EUS image of a focal liver lesion visualized using a linear echoendoscope (Fujifilm EG-580UT) prior to fine-needle biopsy. The hypoechoic lesion is clearly delineated within the hepatic parenchyma.

**Fig 5:**
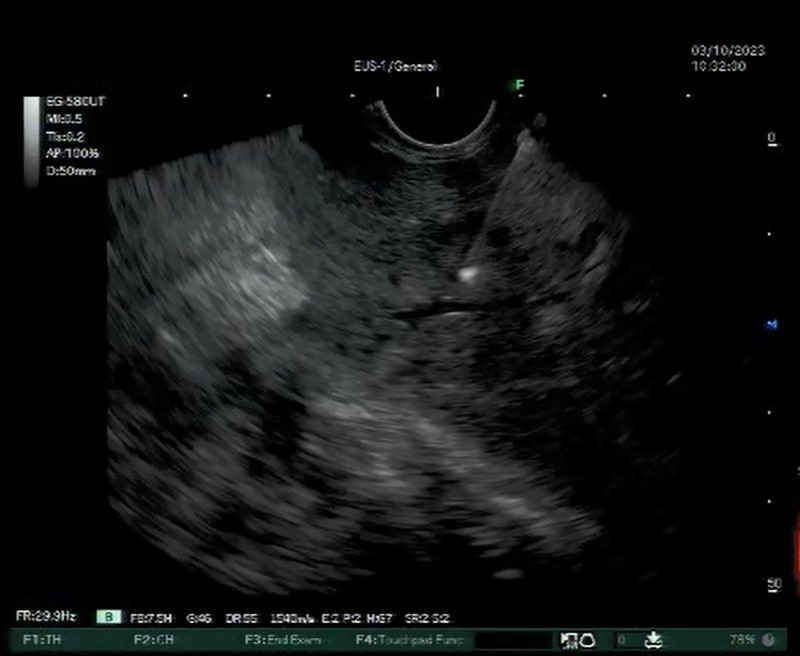
EUS image showing real-time needle placement into a focal liver lesion during EUS-guided fine-needle biopsy. The needle tip is visible within the target lesion.

**Fig 6:**
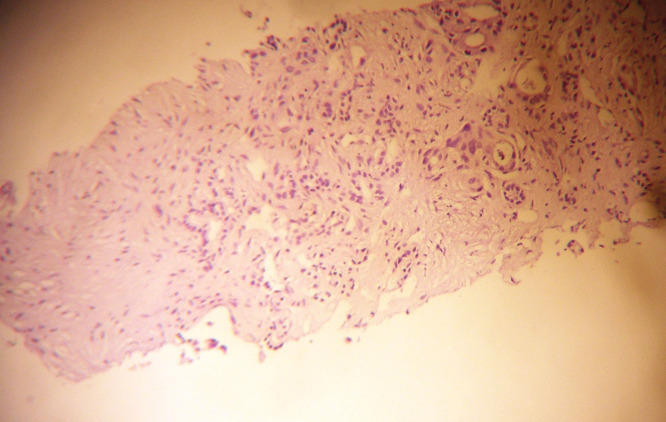
Histopathological specimen (hematoxylin and eosin stain) showing liver tissue with metastatic pancreatic ductal adenocarcinoma obtained by EUS-guided fine-needle biopsy using the 22G Franseen-tip needle.

EUS-FNB of focal liver lesions was performed in 58 (80.5%) patients (Fig 4, Fig 5). Metastases were found in 40 (55.6%) patients: pancreatic ductal adenocarcinoma was detected in 32 (44.4%) (Fig 6), pancreatic acinar cell carcinoma in 1 (1.38%), pancreatic neuroendocrine carcinoma in 5 (6.9%), lung adenocarcinoma in 3 (4.16%), ductal adenocarcinoma of the breast in 2 (2.7%), colorectal adenocarcinoma in 1 (1.38%), Ewing sarcoma in 1 (1.38%), and Hodgkin lymphoma in 1 (1.38%). Primary liver cancer (hepatocellular carcinoma) was diagnosed in 7 (9.7%) patients. Cholangiocellular cancer was found in 3 (4.16%) patients.

There were 3 (4.16%) patients with negative histology for tumor cells: in 2 (2.7%) liver abscess was diagnosed and in 1 (1.38%) hepatic cyst was found. There were no adverse events during the first 48 hours or after the 30-day follow-up. There was no death during the study period. A summary of patient and diagnostic characteristics is presented in Table 1.

**Table 1:** Summary of patient and diagnostic characteristics (N = 72).

Parameter	Malignant (n = 55)	Benign (n = 17)	Total (N = 72)
Median age (years)	62.3	55.3	56.9
Sex (M/F)	23/ 32	9/ 8	32/ 40
Focal lesions	46 (83.6%)	12 (70.6%)	58 (80.5%)
Parenchymal disease	9 (16.4%)	5 (29.4%)	14 (19.4%)
Adverse events	None	None	0%
Technical success	100%	100%	100%

Technical success and specimen adequacy were both achieved in 100% of cases (72/72; exact 95% CI 95.0–100%). Among 72 patients, 55 (76.4%) had malignant and 17 (23.6%) had benign findings.

## Discussion

Determining the etiology of liver disease is based on a variety of diagnostic tools. Serological tests, metabolic markers, and sometimes genetic tests in combination with diagnostic imaging methods are essential for establishing the diagnosis [6]. However, in up to one-third of patients, diagnosis remains unclear, and cases of advanced fibrosis and cirrhosis can be missed with all these methods of investigation. LB plays a major role in assessing the severity of liver disease and is often crucial in determining the diagnosis and its subsequent management [1,2].

LB can be performed via percutaneous (blind or under ultrasonographic or CT control) or transjugular route (preferred in the presence of coagulopathy, large amount of ascites, or high-grade obesity). Surgical (laparoscopic or open) LB is an alternative approach with limited application. Possible complications of percutaneous biopsy include pain, bleeding, infection, peritonitis, and pleural injury with pneumo- or hemopneumothorax. Complications specific to transjugular biopsy include arrhythmias, carotid artery puncture, arterial aneurysms, venous and biliary fistulas, and Horner’s syndrome [3].

Endoscopic ultrasound (EUS) is a well-established imaging modality essential in the evaluation of a broad range of gastrointestinal diseases, including pancreatic, hepatobiliary, and extraluminal processes [3]. The rapid development of EUS in recent years has expanded its application spectrum, allowing precise, real-time, fine-needle biopsies of lesions located near the esophagus, stomach, and duodenum, significantly improving diagnostic accuracy especially in sampling hard-to-reach areas [5].

EUS-guided LB is a relatively new diagnostic method, increasingly proven to be a safe and equivalent alternative to conventional LB over the last two decades. The tool provides high-resolution images of both hepatic lobes, allowing accurate assessment of diffuse liver parenchymal processes and focal lesions [3]. Recent studies have demonstrated the superiority of EUS in the diagnosis and guided biopsy of liver lesions less than 1 cm in size, as well as the higher representativeness of histological material in diffuse parenchymal processes compared with conventional LB [1,2]. This is due to the capability of visualizing and easily accessing both hepatic lobes, reducing variability in histological results caused by the often uneven distribution of pathological processes in the liver parenchyma [3].

EUS-guided LB is typically performed under deep sedation or general anesthesia, which enhances patient comfort compared to percutaneous LB. However, it is important to acknowledge that the requirement for sedation or anesthesia introduces additional risks not present with percutaneous biopsy, which is typically performed under local anesthesia; not all patients may be suitable candidates for deep sedation, and this should be weighed when selecting the biopsy approach. In our cohort, all procedures were performed under procedural deep sedation with propofol, and no patient required general anesthesia. Another advantage of EUS-LB is the significantly shorter postprocedural recovery time (approximately 4 hours) compared to percutaneous LB (at least 10 hours), as well as a lower rate of postprocedural pain [4]. The real-time imaging offered by endoscopic ultrasound facilitates precise targeting of liver tissue, resulting in a safer and more effective biopsy [1,6].

EUS-LB’s endoscopic approach also allows for a simultaneous examination of other abdominal organs, including the pancreas, bile ducts, gallbladder, and lymph nodes, providing comprehensive diagnostic information in a single session and improving efficiency in patient management [5]. In our center, the choice of EUS-LB over percutaneous biopsy is primarily driven by three considerations: the patient already undergoing endoscopy for another indication, allowing tissue acquisition during the same session; lesions that are difficult or impossible to access via a percutaneous approach; and small lesions not reliably targetable percutaneously, for which EUS allows more precise real-time guidance. Biopsies were performed on focal lesions of varying sizes, with a number of procedures also combined with endoscopic evaluation for another indication. Notably, EUS-LB was technically feasible even in lesions as small as 7 mm, which are often difficult to target reliably with a percutaneous approach. EUS-LB, in turn, cannot access all hepatic segments as easily as percutaneous approaches, since the transgastric and transduodenal routes used in EUS-LB primarily allow reliable access to the left lobe and portions of the right lobe. Each approach therefore has complementary blind spots depending on lesion location, consistent with our rationale for selecting EUS-LB when percutaneous targeting was difficult or unfavorable.

Multiple studies in the literature confirm the high diagnostic yield of EUS-LB. It has been demonstrated that EUS-LB with fine-needle biopsy (FNB) needle provides histologically adequate specimens in over 95% of cases [7–9]. The number of passes required to obtain sufficient tissue samples was fewer compared to traditional methods, reducing the risk of complications and procedural time. Overall, complication rates reported were lower than with percutaneous and transjugular LB.

While the initial cost of EUS-LB may be higher than traditional biopsy techniques, the ability to perform multiple diagnostic and therapeutic procedures during a single session can offset costs. Bundling EUS-LB with other interventions such as endoscopic retrograde cholangiopancreatography (ERCP) reduces the need for multiple procedures and hospitalizations, ultimately enhancing cost-effectiveness [10]. It is also important to note that EUS-LB requires specialized equipment, advanced endoscopic expertise, and is typically performed in tertiary referral centers, which may limit its widespread availability compared to percutaneous biopsy. Additionally, our passive surveillance approach to adverse event detection may underestimate the true rate of minor or delayed complications.

In most centers, a 19G core FNB needle is used and has proven its superiority compared to aspiration and tru-cut needles in terms of tissue adequacy and diagnostic accuracy [2,5]. According to multiple comparative studies, higher fragmentation rates, shorter cylinder length, and lower numbers of complete portal tracts are associated with the use of 22G FNB needles [11,12].

The question of whether a smaller needle would achieve comparable diagnostic results was addressed by the prospective study of Hasan et al. involving 40 patients who underwent EUS-LB with a 22G FNB needle (Acquire; Boston Scientific) for elevated liver enzymes. Adequacy of material with subsequent histopathological interpretation was achieved in 100% of cases. Abdominal pain as a postprocedural complication was reported in 15% of cases and underwent complete reversal by 72 hours [9]. Bhat et al. further demonstrated that FNB samples using a 22G needle compared to 19G FNB samples yielded similar total specimen length (24.5 mm vs. 32 mm, p = NS) and similar portal triad counts (20 vs. 21, p = NS), with histologic diagnosis established in the majority of cases with both needle sizes (95%) [13]. More broadly, the shift from fine-needle aspiration toward fine-needle biopsy has been shown to improve histological yield in EUS-guided tissue acquisition generally [14], and a recent systematic review and meta-analysis directly comparing EUS-LB with percutaneous liver biopsy found comparable diagnostic yield between the two approaches [15].

In our center, we routinely use a 22G FNB needle to obtain material echoendoscopically from abdominal tumors (pancreas, enlarged lymph nodes, etc.) to establish a histological diagnosis. Very often, a liver lesion is an incidental finding during the examination, and its biopsy contributes to disease staging.

In our study, we report the results from EUS-LB in 72 patients using a 22G FNB needle and the slow pull technique, demonstrating the efficacy of the method with both high diagnostic yield and an excellent safety profile. All tissue samples were reported as adequate and a diagnosis was established in all patients. However, a discrepancy in diagnostic yield was observed between focal liver lesions, where the material was sufficient to provide a definitive conclusion, and diffuse liver processes, in which the Franseen crown-tip design and smaller needle size likely contributed to a higher fragmentation rate, lower complete portal tract count, and consequently inaccurate fibrosis staging and inability to determine the cause of cholestasis in some cases. In this broader context, evidence directly comparing complete portal tract yield between EUS-LB and percutaneous LB is mixed: a two-center comparative study found comparable portal tract counts between the two approaches (18.5 vs. 21, p = 0.09), although percutaneous biopsy yielded significantly longer specimens [16], and a 2024 meta-analysis of randomized controlled trials similarly found no significant difference in complete portal tract counts between EUS-LB and percutaneous LB, though percutaneous biopsy again achieved a longer maximum specimen length [17].

Our clinical experience demonstrates the high diagnostic value of EUS-LB using a smaller needle, at low peri-interventional risk. Applying the slow pull technique yielded similar diagnostic yield compared to conventional suction techniques, while providing significantly less blood contamination. No adverse events were observed during the first 48 hours or within the 30-day follow-up. Finally, the total cost of the procedure is of importance, especially in economically resource-limited settings where the availability of all needle types may be restricted.

### Limitations

This study has several important limitations. It is a retrospective, single-center study with heterogeneous indications for EUS-LB and an unstandardized decision process; the high proportion of malignant lesions (76.4%) reflects our center’s tertiary referral pattern and should be considered when generalizing our findings. The study also lacks a comparator group (19G FNB, percutaneous, or transjugular biopsy), and we did not compare different suction techniques, needle brands, or assess the correlation between number of passes and specimen adequacy. The diffuse parenchymal disease subgroup (n = 14) was small, and we did not perform a formal cost-effectiveness analysis comparing EUS-LB with conventional biopsy methods. We also did not have independent gold-standard confirmation, such as surgical pathology, for all focal lesions, and more granular histological adequacy parameters were not systematically tabulated for the diffuse parenchymal disease subgroup. Further prospective, comparative studies are warranted to validate these findings.

## Conclusions

Endoscopic ultrasound-guided liver biopsy (EUS-LB) using the 22G fine-needle biopsy (FNB) needle combined with the slow pull technique was associated with a high tissue adequacy rate, favorable diagnostic yield, and a good safety profile in this single-center cohort for diagnosing liver disease. This approach offers significant advantages including precise targeting of liver tissue with minimal fragmentation, fewer complications, and excellent diagnostic yield. The slow pull technique minimizes blood contamination while ensuring adequate tissue samples, making it comparable to traditional suction methods in diagnostic yield. Despite being smaller, the 22G needle provides excellent histological samples with a reduced risk of complications, supporting its potential efficacy, pending confirmation in prospective comparative studies. However, the method has some limitations in diagnostic yield for diffuse liver processes, and further prospective studies are warranted. This method represents a significant advancement in liver biopsy, offering a reliable and patient-friendly alternative to conventional techniques.
